# Serum Soluble Urokinase-Type Plasminogen Activator Receptor: A Promising Biomarker for Stable Chronic Obstructive Pulmonary Disease Patients

**DOI:** 10.7759/cureus.79594

**Published:** 2025-02-24

**Authors:** Rekha D, Priscilla Johnson, Subhasis Das, Sathya GR

**Affiliations:** 1 Physiology, Pondicherry Institute of Medical Sciences, Puducherry, IND; 2 Physiology, Sri Ramachandra Medical College and Research Institute, Sri Ramachandra Institute of Higher Education and Research, Chennai, IND

**Keywords:** glycoprotein, inflammation, prognosis, severity, treatment

## Abstract

Background

Chronic obstructive pulmonary disease (COPD) is a condition in which airflow limitation becomes irreversible over time, often resulting from long-term exposure to environmental pollutants, harmful particles, smoke, and biomass fuel. Beyond FEV1, identifying a more specific biomarker to predict COPD progression remains a challenge. Soluble urokinase-type plasminogen activator receptor (suPAR) expression increases in the respiratory epithelial cells of COPD patients. This study aimed to evaluate serum suPAR levels across different grades of stable COPD patients.

Methods

Two hundred stable COPD patients (148 males and 52 females) were recruited after obtaining informed consent. Blood samples were collected, and serum suPAR levels were measured in all participants.

Results

Serum suPAR levels were elevated in COPD patients at Global Initiative for Obstructive Lung Disease (GOLD) stages IV and III (6.38 ± 0.05 ng/ml and 5.82 ± 0.18 ng/ml, respectively) compared to those at GOLD stages II and I (5.15 ± 0.25 ng/ml and 4.17 ± 0.29 ng/ml). A one-way ANOVA confirmed that the differences between groups were statistically significant (F = 428.83, p < 0.001).

Conclusions

This study suggests that serum suPAR levels can serve as a diagnostic marker for COPD. As low-grade pulmonary inflammation increases with disease severity, suPAR levels also rise. Additionally, this marker may be useful for monitoring the prognosis of stable COPD and assessing treatment response.

## Introduction

Chronic obstructive pulmonary disease (COPD) is the second leading cause of mortality among noncommunicable diseases in India. It is characterized by airflow limitation resulting from persistent low-grade pulmonary inflammation triggered by various environmental factors. Major risk factors for COPD include smoking, occupational dust, air pollution, and biomass exposure. Prolonged exposure to these harmful particles damages lung parenchymal tissue and disrupts essential defense mechanisms, such as mucociliary clearance and phagocytosis, increasing susceptibility to infections and exacerbations. This disruption of lung defense mechanisms contributes to premature mortality among exposed individuals compared to those not exposed. The primary causes of premature death in COPD patients include respiratory insufficiency and cardiovascular complications, both of which are driven by oxidative stress and systemic inflammation. These processes impair macrophage-mediated clearance of apoptotic cells, thereby limiting tissue repair [1,2].

Traditionally, COPD has been diagnosed based on patient history, clinical examination, and spirometry, as no standardized tests or specific biomarkers for lung inflammation have been established. This highlights the need for more accurate and accessible biomarkers to improve COPD diagnosis, prognosis assessment, and disease management, ultimately reducing the health and economic burden associated with COPD.

Several biomarkers, such as CRP, fibrinogen, and gamma-glutamyl transferase, are commonly used to assess systemic inflammation [3-5]. However, these markers are not reliable for predicting disease prognosis and mortality. In contrast, soluble urokinase-type plasminogen activator receptor (suPAR) is a more stable biomarker that can indicate disease morbidity in individuals with low-grade inflammation [6,7]. The presence of suPAR in a patient’s serum reflects immunological dysfunction in the lungs and provides a better prognostic value for underlying inflammation compared to nonspecific markers [8-11].

Clinically, suPAR has been used to diagnose conditions such as systemic inflammatory response syndrome, cancer, focal segmental glomerulosclerosis, cardiovascular disease, type 2 diabetes, and infectious diseases, including HIV [12]. While COPD is a major cause of mortality among noncommunicable diseases, research on suPAR in COPD patients is limited in India, despite its established role in predicting disease severity and prognosis in Western countries [1,6]. Therefore, the present study aimed to measure serum suPAR levels in stable COPD patients and examine its correlation with disease severity.

## Materials and methods

This cross-sectional study was conducted among 200 stable COPD patients aged 35 to 65 years. The sample size was estimated based on previous research, which reported a correlation of -0.478 between suPAR and FEV1% of predicted (sample correlation) and -0.3 in the population, with a power of 80%, an alpha error of 5% for a two-sided hypothesis, and a 10% attrition rate. The final sample size was determined to be 200 using N Master software (Christian Medical Centre, Vellore, India) [6].

Patients with a confirmed history of COPD were included in the study. After obtaining institutional ethical clearance (RC/17/32), the study was conducted between January 2018 and March 2018. Patients with a history of bronchial asthma, tuberculosis, diabetes, cancer, renal disease, or collagen tissue disease were excluded, as these conditions can alter suPAR levels.

COPD diagnosis was confirmed through spirometry by assessing the post-bronchodilator FEV1/FVC ratio. Based on FEV1% predicted, disease severity was classified according to the Global Initiative for Obstructive Lung Disease (GOLD) criteria. Mild COPD (GOLD I) was defined as FEV1 ≥80% of predicted, moderate COPD (GOLD II) as FEV1 ≥50% but <80% of predicted, severe COPD (GOLD III) as FEV1 ≥30% but <50% of predicted, and very severe COPD (GOLD IV) as FEV1 <30% of predicted [13].

Following spirometry, blood samples were collected from each stable COPD patient between 9 AM and 12 PM. Fasting was not required for sample collection. Serum was separated and stored at -20°C in a deep freezer. Serum suPAR levels were measured using an EIAab kit (EIAab Science Co., Ltd., Wuhan, China), with a sensitivity of <0.022 ng/mL, an intra-assay coefficient of variance of <4.4%, and an inter-assay coefficient of variance of <8.3%.

Reagents, samples, and standards were prepared according to the manufacturer's instructions. A total of 100 µL of the standard was added to the samples in each well, followed by incubation for 2.5 hours. Then, 100 µL of biotin antibody was added to each well and incubated for one hour at room temperature. After four washes, 100 µL of streptavidin solution was added and incubated for 45 minutes. The plates were washed again four times before adding 100 µL of TMB One-Step Development Solution, which was incubated for 30 minutes at room temperature. Finally, 50 µL of Stop solution was added to each well, and the optical density was measured at 450 nm using colorimetry.

Statistical analysis

The data were entered into Microsoft Excel (Microsoft Corporation, Redmond, WA, USA) and analyzed using IBM SPSS Statistics for Windows, Version 21.0 (Released 2012; IBM Corp., Armonk, NY, USA). Descriptive analysis, including mean and SD, was performed. The mean and SD of serum suPAR levels for different grades of stable COPD were calculated. Differences in mean suPAR values across the various COPD severity grades were assessed using ANOVA.

## Results

Demographic data, including age, height, weight, BMI, pack-year index, partial pressure of oxygen, and FEV1/FVC% predicted, are presented in Table 1. The mean ± SD of FEV1% predicted for COPD patients was calculated, as shown in Table 2. Serum suPAR levels across different COPD severity grades were determined and are presented in Table 3. The mean serum suPAR values increased with higher COPD severity grades. A one-way ANOVA revealed a significant difference between the groups (F = 428.83; p < 0.001*). 

**Table 1:** Mean and SD values of age, height, weight, BMI, pack-year index, SpO₂, and FEV1/FVC of COPD patients COPD, chronic obstructive pulmonary disease

Demographic parameter	Mean	SD
Age (years)	50.24	8.53
Height (cm)	157.45	6.25
Weight (kg)	57.3	8.26
BMI (kg/m²)	22.96	2.7
Cigarette (pack-years)	10.23	7.32
SpO₂	92.79	2.83
FEV1/FVC (% predicted)	49.64	12.78

**Table 2:** Mean and SD of FEV% predicted for COPD COPD, chronic obstructive pulmonary disease

COPD grade (FEV1% predicted)	Number of patients (frequency %)	FEV1% predicted mean	FEV1% predicted SD
I	10 (5%)	83.1	2.13
II	44 (22%)	60.02	6.77
III	124 (62%)	40.21	5.46
IV	22 (11%)	27.9	1.02

**Table 3:** Mean ± SD serum suPAR levels among different grades of COPD ^* ^Statistically significant COPD, chronic obstructive pulmonary disease; GOLD, Global Initiative for Obstructive Lung Disease; suPAR, soluble urokinase-type plasminogen activator receptor

Serum biomarker	GOLD stage classification of COPD (mean ± SD)				p-Value
	I (n = 10)	II (n = 44)	III (n = 124)	IV (n = 22)	
suPAR (ng/ml)	4.17 ± 0.29	5.15 ± 0.25	5.82 ± 0.18	6.38 ± 0.05	<0.001^*^

## Discussion

Urokinase-type plasminogen activator receptor (uPAR) is normally kept inactive by plasminogen activator inhibitors [14,15]. However, exposure to air pollutants or smoke activates the urokinase plasminogen activator, which stimulates the respiratory epithelium [16-19]. The soluble and active portion of the receptor, known as suPAR, enters the bloodstream, triggering plasminogen, inactivating anti-proteases, and inhibiting the phagocytic activity of alveolar macrophages. This cascade ultimately leads to extracellular matrix degradation and airway destruction [20,21].

In the present study, serum suPAR levels were significantly higher in grade IV COPD patients compared to those in grades I-III. Similar findings have been reported in previous studies, which showed elevated suPAR levels in grade IV COPD compared to lower-grade COPD. The increase in suPAR levels has been attributed to the upregulation of uPAR expression in the respiratory epithelium. As COPD severity progresses, the number of uPARs increases, further amplifying pulmonary inflammation and airflow limitation [20].

In this study, suPAR demonstrated a statistically significant negative correlation with FEV1%. The probable explanation is that prolonged exposure to risk factors leads to increased urokinase plasminogen activator receptor expression, resulting in elevated suPAR levels. This, in turn, triggers airway remodeling and airflow limitation, contributing to a decline in spirometry parameters. Similar findings have been reported in previous studies, which observed a decline in lung function, particularly FEV1%, with increasing suPAR levels (r = -0.6; p < 0.01) and (r = -0.478; p = 0.001). Studies by Wang et al. and Celli et al. have also demonstrated a negative correlation between serum suPAR and spirometry parameters such as FEV1% [22,23].

Although smoking, age, and gender were considered potential confounding factors, suPAR levels were not statistically significant in relation to these variables (p = 0.8, p = 0.4, and p = 0.5, respectively).

Limitations

The present study is cross-sectional, which limits the ability to assess disease progression. Additionally, the number of study participants was uneven across different COPD grades. The study did not include a control group or assess suPAR levels in patients experiencing acute COPD exacerbations. Furthermore, a detailed drug history was not obtained, which could have influenced serum suPAR levels.

## Conclusions

The results of the present study indicate that serum suPAR can be used to assess COPD severity. While pulmonary function tests such as spirometry and the six-minute walk test are available, they are subjective, effort dependent, and may be influenced by adaptation over time. To overcome these limitations, serum suPAR, as a quantitative marker, could serve as a more reliable indicator of disease severity in the future. However, prospective cohort studies are needed to further evaluate the role of suPAR in disease prognosis. Such research could contribute to improved clinical outcomes and help reduce both the disease burden and associated economic costs.
